# Progress with COVID vaccine development and implementation

**DOI:** 10.1038/s41541-024-00867-3

**Published:** 2024-04-01

**Authors:** Richard W. Titball, David I. Bernstein, Nicolas V. J. Fanget, Roy A. Hall, Stephanie Longet, Paul A. MacAry, Richard E. Rupp, Marit van Gils, Veronika von Messling, David H. Walker, Alan D. T. Barrett

**Affiliations:** 1 Department of Biosciences, University of Exeter, Devon, UK; 2 Cincinnati Children’s Hospital Medical Center, University of Cincinnati, Cincinnati, OH USA; 3 Nature Research, London, UK; 4 Australian Infectious Diseases Research Centre, School of Chemistry and Molecular Biosciences, The University of Queensland, St Lucia, QLD Australia; 5 CIRI - Centre International de Recherche en Infectiologie, Team GIMAP, Univ Lyon, Université Claude Bernard Lyon 1, Inserm, U1111, CNRS, UMR5308, CIC 1408 Vaccinology, F42023 Saint-Etienne, France; 6 Department of Microbiology and Immunology, Yong Loo Lin School of Medicine, National University of Singapore, Singapore, Singapore; 7 Department of Pediatrics, University of Texas Medical Branch, Galveston, TX USA; 8 Department of Medical Microbiology and Infection Prevention, Amsterdam Institute for Immunology and Infectious Diseases, Amsterdam UMC, University of Amsterdam, Amsterdam, The Netherlands; 9 Federal Ministry of Education and Research, Berlin, Germany; 10 Department of Pathology, University of Texas Medical Branch, Galveston, TX USA; 11 Sealy Institute for Vaccine Sciences and Department of Pathology, University of Texas Medical Branch, Galveston, TX USA

January 2024 marks the fourth anniversary of the identification of SARS-CoV-2 as the causative agent of the COVID-19 epidemic. Since that time, vaccine development has proceeded at an unprecedented and extraordinary pace due to the combined efforts of researchers from academia, industry, government and non-governmental organisations. These efforts, together with regulators, enabled the first vaccines to receive Emergency Use Authorisation (or equivalent) in under 12 months. As of February 2024, 64 vaccines are approved by one or more national regulatory authorities^1^. Vaccine platforms include inactivated viruses, mRNA, DNA, recombinant proteins, non-replicating and replicating viral vectors. Immunogens include the spike protein, receptor-binding domain, and spike protein ectodomain. Whilst the estimates of annual global deaths linked to COVID-19 have reduced by as much as 95% since the start of the pandemic, the disease remains a significant public health concern, with up to two million new infections reported annually. These new infections are linked to the emergence of variants of the original Wuhan-Hu-1 strain of SARS-CoV-2 that encode mutations that decrease the effectiveness of pre-existing immunity.

*NPJ Vaccines* has now published over 200 papers on COVID-19 vaccines across a broad range of areas, from basic science to next-generation vaccines and the implementation of vaccine programmes. In this Editorial, we provide a brief overview of 34 selected publications on *NPJ Vaccines* over the past 2 years, which illustrate the challenges faced and the progress made.

The rapid pace of vaccine research and development is highlighted in the review article authored by Kyriakidis et al. in early 2021. Although only a year after the sequencing of the SARS-CoV-2 genome, there were already 63 candidate SARS-CoV-2 vaccines in clinical trials, 13 of which were in, or entering into, phase III clinical trials^2^. All vaccines assessed in phase III produced neutralising antibodies, with mRNA vaccines inducing the highest antibody titres^3^. In 2023 Zhang et al.^4^ reported that the Moderna (mRNA-1273) and Pfizer-BioNTech (BNT162b2) mRNA vaccines, formulated in different lipid nanoparticles, showed differences in both stability at 4 °C and antibody production in mice. mRNA vaccines can be found in tissues such as axillary lymph nodes for at least 30 days post-immunisation^5^ leading to the induction of T-cell responses with a Th1 bias.

Whilst the immune responses to vaccines have been a focus of attention, we have seen several publications documenting the side effects of COVID-19 vaccines. Most of these side effects are minor and controlled using analgesics which appear to have no significant impact on antibody levels or vaccine efficacy in adults, with inconclusive evidence of effects in children^6^. Other side effects of vaccination could be argued to have unintentional benefits. For example, Gyöngyösi et al.^6^ reported that SARS-CoV-2 vaccination reduced the rate of DNA virus activation in patients with long-COVID syndrome, possibly by interrupting viral cross-talk. Serious adverse events, such as retinal vascular occlusion, also occur but are rare, occurring up to 2 years after vaccination with a hazard ratio of 2.19 compared to matched COVID-19 naïve unvaccinated individuals^7^, but need to be viewed against the background of thrombotic events associated with SARS-CoV-2 infection.

Information about the roles of humoral and cellular immunity in protection continues to emerge. Heinz and Stiansy^8^ reviewed the differences in spike(S)-protein presentation to the immune system by different COVID-19 vaccines and how this likely influences the immune response. Levels of circulating antibodies to the S-protein, able to neutralise the virus, are known to correlate with protection^9^. However, there is a paucity of data on the comparative efficacy of different vaccines. The calibration of neutralising antibody levels against World Health Organisation (WHO) standards allows a readout as International Units enabling comparisons, something this journal has required since 2022. Using this approach, Karbiener et al.^10^ found that neutralising antibody levels induced by the Astra-Zeneca vectored vaccine (Vaxzevria) were statistically lower than the levels of neutralising antibodies induced by the Pfizer-BioNTech mRNA vaccine or an adjuvanted S-protein nanoparticle vaccine produced by Novavax (NVX-CoV2373), which were statistically indistinguishable from each other.

T-cells also play a critical role in protection and cross-protection against variants. After vaccination with a Wuhan-Hu-1 derived DNA vaccine, both CD4+ and CD8+ T-cells were able to protect mice against Gamma or Omicron variants in the absence of significant levels of neutralising antibodies^11^. Intranasal vaccines have the potential to broaden the immune response and provide improved protection of mucosal surfaces by inducing local IgA and tissue-resident T-cell responses^12^. As our understanding of mechanisms of protection increases, efforts have also been directed toward understanding the efficacy of vaccine responses in individuals with co-morbidities. For example, individuals with HIV being treated with antiretroviral therapy and with normal CD4+ T cell counts can mount neutralising antibody responses after two, but not after one, vaccine dose^13^.

One focus of vaccine research in the past 2 years has been to identify ways of enhancing the efficacy of vaccines by increasing the magnitude and longevity of protection. Improved efficacy should also reduce virus transmission; an increasingly important factor as the R_0_ of variants increased compared to the original Wuhan-Hu-1 virus. We have seen a number of important studies published in *NPJ Vaccines* that explore different approaches to address these challenges. Immunisation with a prefusion-stabilised S-protein trimer enhanced antibody responses compared to the monomer^14^ while delaying the second booster dose of mRNA vaccines enhanced the antibody (but not the cellular) immune response^15^. The role of adjuvants in improving immunogenicity of subunit^16,17^ or mRNA^18^ vaccines has been a focus of several investigations. Another approach has been to deliver vaccines at mucosal sites with the hope that virus transmission can be dramatically reduced and protection of the immunised individual improved^19,20^. The opportunities and challenges associated with mucosal vaccination were concisely summarised in the report of a meeting organised by the National Institute of Allergy and Infectious Diseases (NIAID), The Coalition for Epidemic Preparedness Innovation (CEPI), The Bill & Melinda Gates Foundation (BMGF), The Biomedical Advanced Research and Development Authority (BARDA), and the Wellcome Trust^21^.

The emergence of SARS-CoV-2 variants, especially the Omicron lineage, resulted in reduced immune protection elicited from previous vaccination or natural infection and raised concerns over the efficacy of current vaccines. Consequently, there has been a surge of studies investigating the immunological cross-reactivity to emerging variants. The fact that individuals, especially children^22^, can develop immune responses that cross-react against newly emerging variants provides optimism that universal vaccines may be feasible. Both hybrid immunity and rapid booster vaccination offer the potential for improved protection against variants^23,24^. This possibility was confirmed by Lyke et al.^25^, who showed that protein-based booster vaccination with NVX-CoV2373 in individuals who had been primed with mRNA or viral vector vaccines resulted in increased antibody levels against different variants and especially in individuals previously infected with SARS-CoV-2. Another approach involves using S-protein modified to include mutations found in different variants to induce cross-protective immunity^14^. Zhao et al.^26^ elegantly summarised the challenges associated with designing universal vaccines and possible solutions, including heterologous prime-boost regimens, construction of chimeric immunogens, design of protein nanoparticle antigens, and utilisation of conserved neutralising epitopes. The heterologous prime-boost vaccination study with BA.1 or BA.4/BA.5 reported by Springer et al.^27^ indicates that this strategy results in increased antibody titres against matched heterologous variants but is less effective towards newly emerged variants, such as XBB.1.5. This finding emphasises the need for creative novel solutions to elicit more broad protection or reduce the number of repeated boosters. New strategies could include coadministration of vaccines against other viruses, as elegantly demonstrated by Ye et al.^28^, who showed protection using a SARS-CoV-2 and influenza combined mRNA vaccine.

The risk of local epidemics or large-scale pandemics from newly emerged agents has increased over the past century, and even though it is difficult to identify the pathogens that will cause the next outbreak/pandemic, most viral pandemics in the 20th century were due to zoonotic spillover. Thus, it is crucial to develop better global pandemic preparedness measures based on lessons learned from the COVID-19 response. Wiliams et al.^29^ outline the range of interventions, including vaccine development and deployment strategies, which are likely to be key, but which may be shaped by socio-structural forces. Investment in early-stage vaccine platforms is crucial. One focus should be on strategies addressing current vaccine limitations related to cross-protective immunity, large-scale distribution, and vaccine storage. For instance, self-amplifying RNA vaccines that are stable at room temperature could be developed^30^. Secondly, it is essential to sustain and develop robust vaccine manufacturing capabilities. India demonstrated that using existing production, manpower and cold-chain infrastructure, it was able to quickly expand its vaccine manufacturing capacity, to produce both domestic and foreign-based vaccines and to lead vaccine development and deployment programmes^31^. Thirdly, improving coordination and establishing agreements between countries, and vaccine industries, is needed to improve the global distribution of vaccines, including to low-income countries^32^. Finally, the success of a vaccine programme is also based on the vaccine acceptance rate. A study by Kreps et al. in 2020^33^ demonstrated that Emergency Use Authorisation had a negative effect on the willingness of individuals to be vaccinated, but this view changed over time. Consequently, it is valuable to develop longitudinal and methodological approaches to understand the factors which impact vaccine attitudes and behaviours, including how politicised COVID-19 vaccine spillover in the USA might have shaped views towards vaccines^34^.

In summary, COVID-19 vaccines have been shown to be safe and effective in most individuals and have played a major role in controlling the pandemic. The pandemic has driven the evolution of new vaccine technologies, which are now finding applications in the control of other infectious and non-infectious diseases. However, there is still a need to improve vaccine-induced protection, reduce the shedding and transmission of viruses, and enhance the ability of vaccines to provide broader protection against new variants of emerging pathogens. Our improved understanding of the roles of different arms of the immune system in protective immunity will underpin this work, and we are starting to see tangible progress in this area. Equally important, the COVID-19 vaccination programme has highlighted a wide range of issues that require urgent attention in order to prepare for future outbreaks of other infectious diseases. Whilst the initial focus of COVID-19 vaccine programmes was to identify vaccine candidates, the challenges of scaling up vaccine production, distributing vaccines, understanding public acceptance of vaccines, and ensuring a coordinated global approach initially received less attention than necessary. However, ultimately these became major challenges to the effective control of the pandemic. We have made a good start to pandemic preparedness but still have some way to go to ensure we are prepared for the next pandemic.
